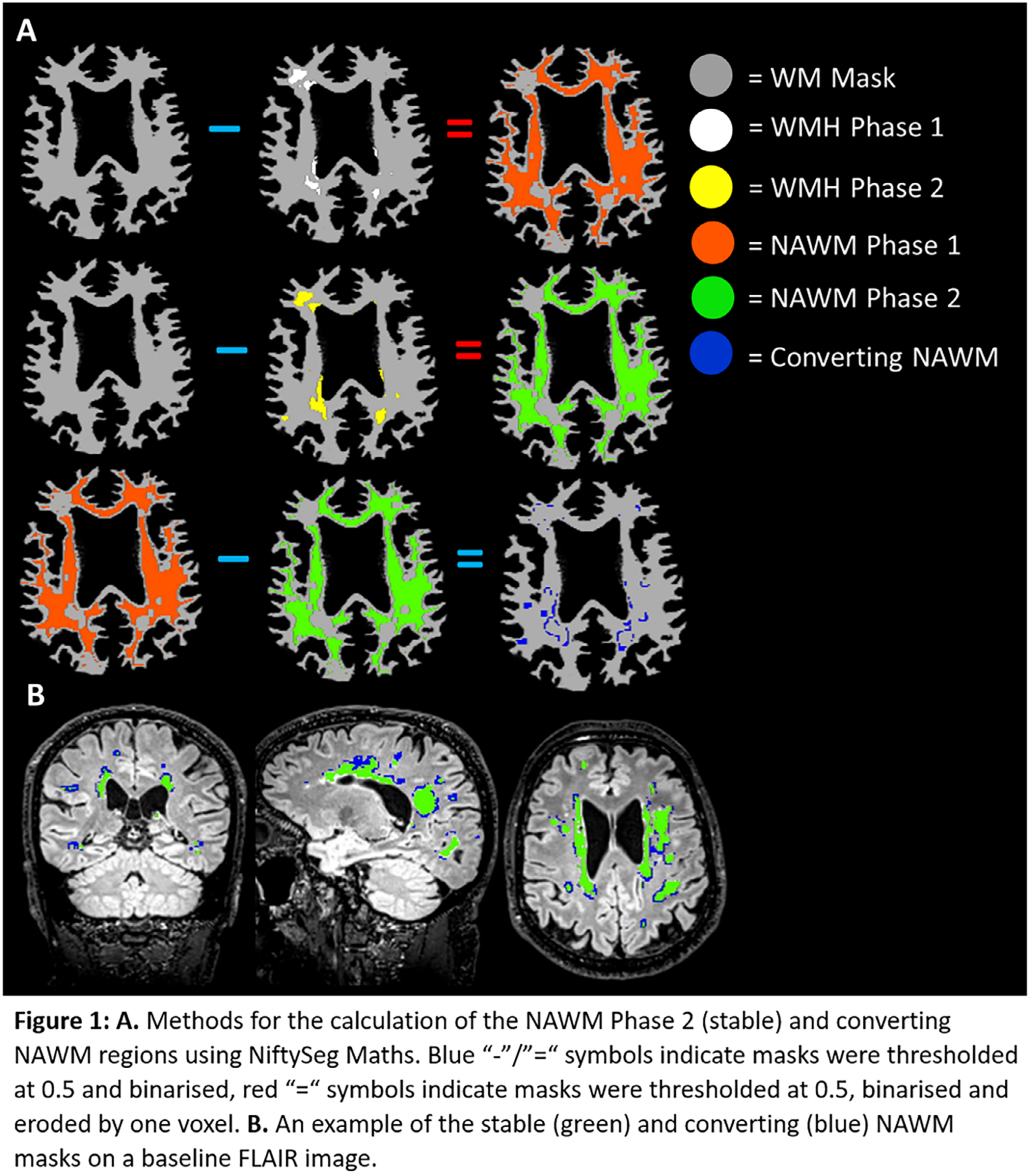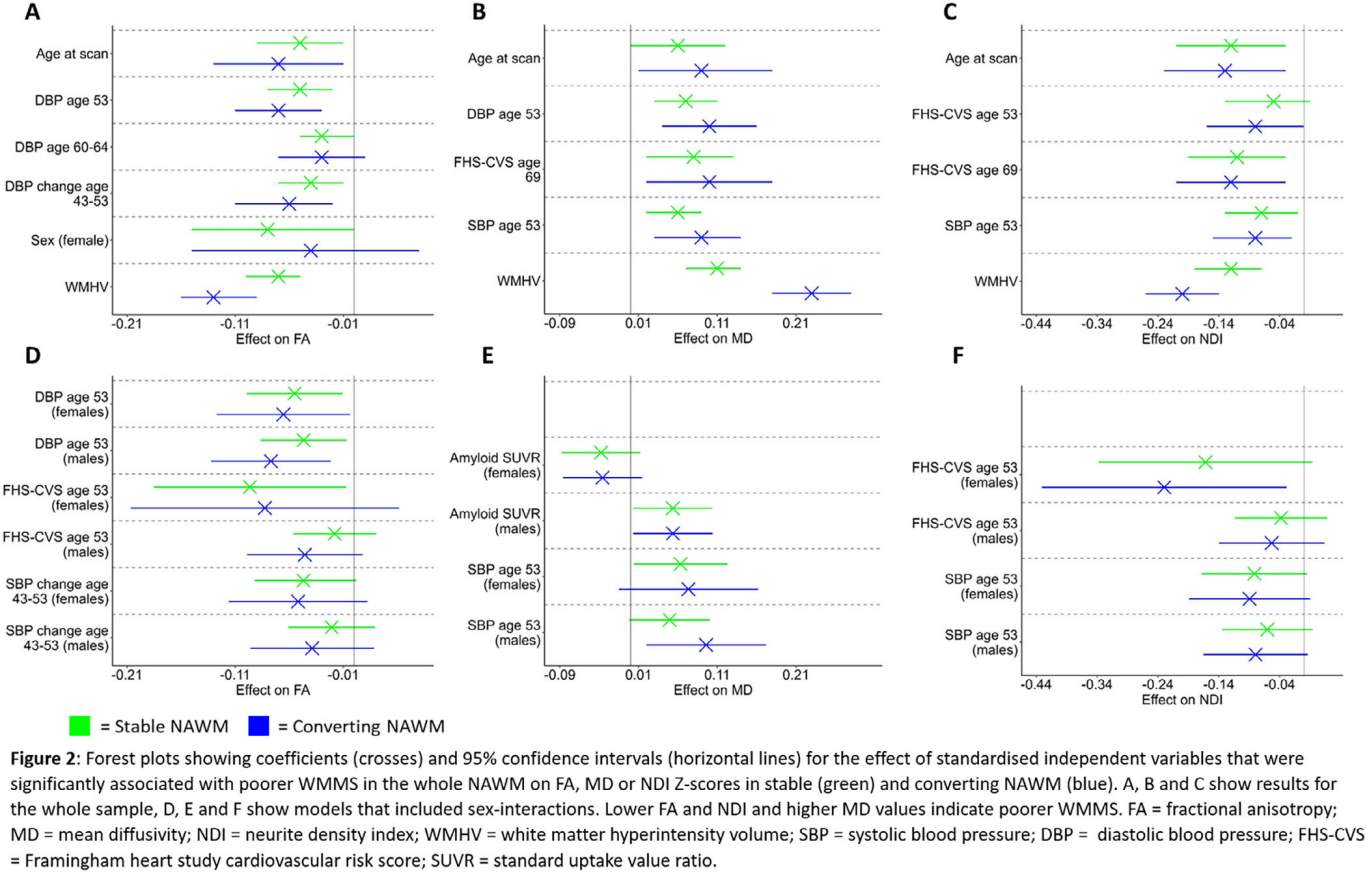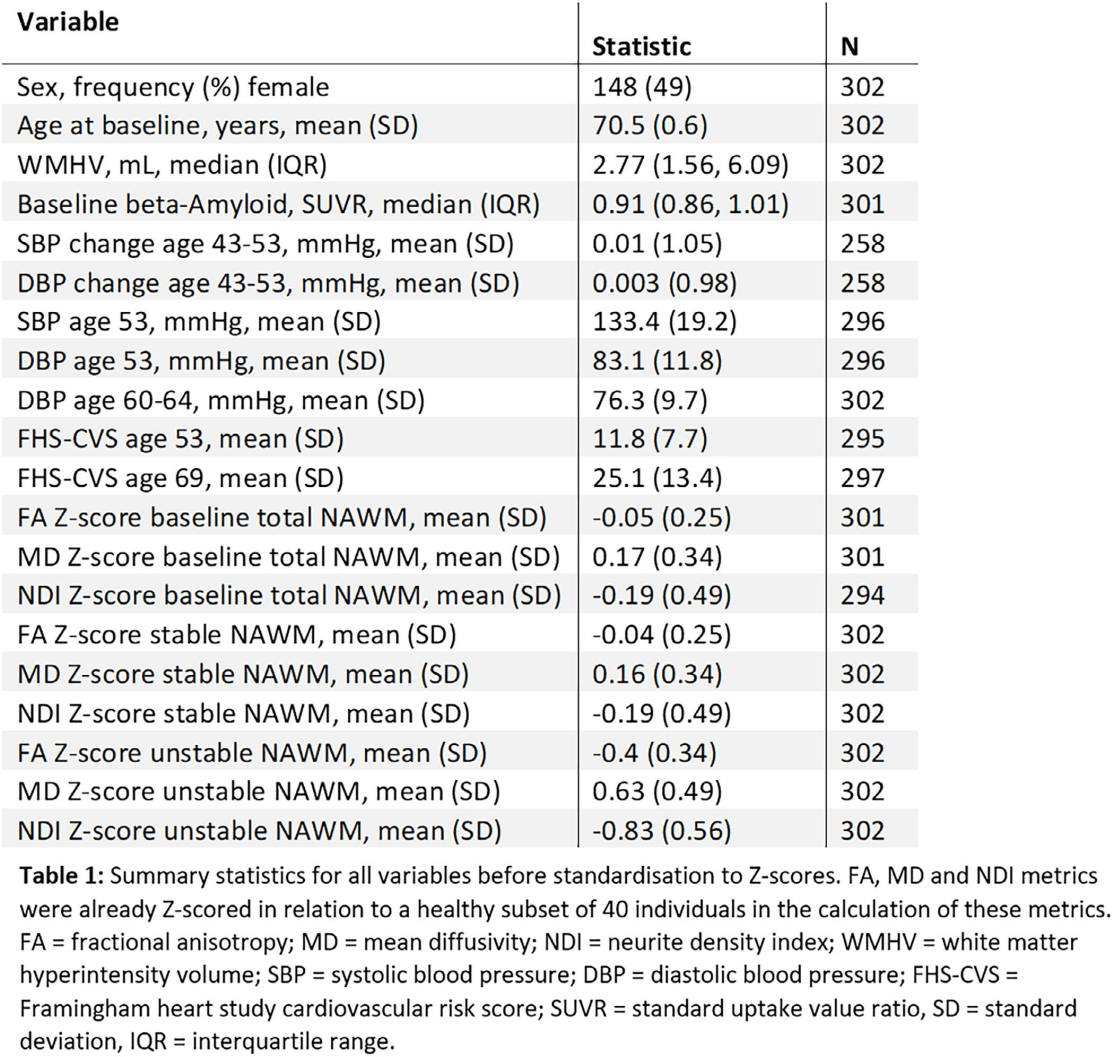# Investigating the effects of brain and cardiovascular health on white matter microstructural properties: a comparison of stable and converting normal appearing white matter

**DOI:** 10.1002/alz70856_104402

**Published:** 2025-12-26

**Authors:** Thomas M. Brown, Sarah‐Naomi James, Jennifer M Nicholas, Ian B. Malone, William Coath, Heidi Murray‐Smith, David M Cash, Frederik Barkhof, Jonathan M Schott, Jo Barnes, Carole H Sudre

**Affiliations:** ^1^ Dementia Research Centre, UCL Queen Square Institute of Neurology, University College London, London, United Kingdom; ^2^ Medical Research Council Unit for Lifelong Health and Ageing at UCL, London, United Kingdom; ^3^ Department of Medical Statistics, London School of Hygiene and Tropical Medicine, London, United Kingdom; ^4^ UK Dementia Research Institute at UCL, London, United Kingdom; ^5^ Hawkes Institute, University College London, London, United Kingdom; ^6^ Department of Radiology and Nuclear Medicine, Vrije Universiteit Amsterdam, Amsterdam UMC, Amsterdam, Netherlands; ^7^ MRC Unit for Lifelong Health and Ageing, University College London, London, United Kingdom; ^8^ School of Biomedical Engineering and Imaging Sciences, King's College London, London, United Kingdom

## Abstract

**Background:**

Diffusion weighted imaging (DWI) measures white matter (WM) microstructure (WMMS), with changes in various DWI metrics seen in Alzheimer's disease (AD) progression^1^. Using data from Insight46^3–5^, a British population‐based birth cohort, we previously found poorer WMMS in normal appearing WM (NAWM) age 70 had sex‐dependent associations with higher midlife blood pressure and cardiovascular risk, as well as concurrent WM hyperintensity volume (WMHV) and β‐Amyloid (Aβ) burden^2^. Here we explore whether these findings reflect poorer WMMS in areas of NAWM that remained normal appearing ∼2.5 years later, or in areas that later became WMH.

**Method:**

Using data from *N* = 302 Insight46 participants who were scanned for a second time ∼2.5 years later, we used linear regression to re‐examine the previously identified significant relationships with NAWM integrity^2^, as well as sex interactions in men and women that remained robust in the smaller follow‐up sample, separately in “stable” (remained NAWM) and “converting” (converted to WMH) regions (Figure 1). WMMS metrics were fractional anisotropy (FA), mean diffusivity (MD) and neurite density index (NDI)^2^. Analyses were adjusted for sex and age as well as total intracranial volume (TIV) for models with WMHV and Aβ.

**Result:**

Summary statistics are shown in Table 1. The associations shown in the total NAWM^2^ persisted in stable regions (Figure 2). Stronger associations were observed in the converting NAWM, particularly with WMHV at age 70. There was also a significant association between BP at age 53 and FA and MD in males in the converting NAWM, which was only observed in women in the whole region^2^. The effect of Aβ on MD in men was robust in both regions.

**Conclusion:**

Previously demonstrated associations with poorer WMMS (age, midlife cardiovascular risk factors, WMHV and Aβ)^2^ were not driven solely by NAWM on the verge of becoming WMH. However, stronger effects observed in converting NAWM suggest higher midlife BP, poorer WMMS and WMH development are part of a continuous, related process, particularly in men. The similar effect of Aβ burden in men in the stable and unstable NAWM suggests the effects of Aβ on WMMI are not driven by tissue later converting to WMH.